# Gender Difference in Architectural and Mechanical Properties of Medial Gastrocnemius–Achilles Tendon Unit In Vivo

**DOI:** 10.3390/life11060569

**Published:** 2021-06-17

**Authors:** Liqin Deng, Xini Zhang, Songlin Xiao, Baofeng Wang, Weijie Fu

**Affiliations:** 1 School of Kinesiology, Shanghai University of Sport, Shanghai 200438, China; 1921516007@sus.edu.cn (L.D.); 1911516024@sus.edu.cn (X.Z.); 1821517025@sus.edu.cn (S.X.); 1821516025@sus.edu.cn (B.W.); 2 Key Laboratory of Exercise and Health Sciences of Ministry of Education, Shanghai University of Sport, Shanghai 200438, China

**Keywords:** gender, medial gastrocnemius, Achilles tendon, architecture, mechanical properties

## Abstract

This study aims to explore whether gender differences exist in the architectural and mechanical properties of the medial gastrocnemius–Achilles tendon unit (gMTU) in vivo. Thirty-six healthy male and female adults without training experience and regular exercise habits were recruited. The architectural and mechanical properties of the gMTU were measured via an ultrasonography system and MyotonPRO, respectively. Independent *t*-tests were utilized to quantify the gender difference in the architectural and mechanical properties of the gMTU. In terms of architectural properties, the medial gastrocnemius (MG)’s pennation angle and thickness were greater in males than in females, whereas no substantial gender difference was observed in the MG’s fascicle length; the males possessed Achilles tendons (ATs) with a longer length and a greater cross-sectional area than females. In terms of mechanical properties, the MG’s vertical stiffness was lower and the MG’s logarithmic decrement was greater in females than in males. Both genders had no remarkable difference in the AT’s vertical stiffness and logarithmic decrement. Gender differences of individuals without training experience and regular exercise habits exist in the architectural and mechanical properties of the gMTU in vivo. The MG’s force-producing capacities, ankle torque, mechanical efficiency and peak power were higher in males than in females. The load-resisting capacities of AT were greater and the MG strain was lesser in males than in females. These findings suggest that males have better physical fitness, speed and performance in power-based sports events than females from the perspective of morphology and biomechanics.

## 1. Introduction

The medial gastrocnemius–Achilles tendon unit (gMTU) is the largest but most vulnerable muscle–tendon unit in the body [1,2]. On the one hand, the gMTU plays an essential role in producing force, as well as in storing and releasing mechanical energy, for supporting the body weight and propulsion in the stance phase during walking, jumping or running [1,3]. On the other hand, calf pain or strain and Achilles tendon (AT) injuries commonly occur and cost a lot [4,5,6]. Interestingly, the architectural and mechanical properties of the gMTU, such as the stiffness and length of the AT and the fascicle length and pennation angle of the medial gastrocnemius (MG), reflects physical fitness and exercise performance, as well as susceptibility to injuries [7,8,9,10,11,12].

Based on previous studies, gender difference is found in physical fitness and exercise performance. Males have higher overall physical fitness z-score values [13] and better performance compared with females, especially in testing items or sports events related to the strength of lower extremity, speed and endurance (muscle strength, ankle moment/power, dynamic stability, running economy, sprint performance, etc.) [14,15,16] Physical function plays an important role in overall health [17]; thus, the mechanism that causes gender difference should be studied. Whether or not the architectural and mechanical properties of the gMTU are a biomechanical mechanism of the superior physical fitness and exercise performance in males was explored in this study. Exercise or training methods can possibly enhance the specific architectural and mechanical immerits of the gMTU in females to remedy inferior physical fitness and exercise performance.

The incidence of musculoskeletal disorders, such as foot pain, Achilles tendinopathy or patellofemoral pain syndrome, is higher in females compared with males [18]. However, the occurrences of AT and calf injuries are somewhat greater in males than in females [18,19]. The inconsistency could be partly attributed to the inclusion criteria of subjects. For example, Kvist et al. [19] believed that the higher rate of participation in sports by males is the reason for the uneven injury rate. The pure gender factor that affects the incidence of MG and AT injuries is still unclear. Thus, males and females, without training experience and regular exercise habits, were recruited in this study to provide references for understanding the gender difference in the incidence of MG and AT injuries.

The gender difference in the architectural and mechanical properties of the gMTU of Asian individuals are poorly studied. Therefore, this study aimed to explore whether gender differences exist in the architectural and mechanical properties of the gMTU in vivo. We hypothesized that: (1) for architectural properties, the MG’s fascicle length, pennation angle and thickness are greater or longer in males than in females; the AT’s length and cross-sectional area (CSA) are longer or greater in males than in females; (2) concerning mechanical properties, males possessed greater MG and AT vertical stiffness than females, and females had a higher logarithmic decrement of MG and AT.

## 2. Materials and Methods

### 2.1. Participants

Eighteen healthy male and eighteen healthy female Asian adults with no training experience and regular exercise habits were recruited (Table 1). The sample size was calculated by G*power (Version 3.1.9.6, Kiel University, Kiel, Germany). The effect size was calculated using the results of the comparison of the MG’s pennation angles between males and females in the study of Chow et al. [14] The results showed that 18 participants in each group was enough (effect size: 0.99, significance level: 0.05, statistical power: 80%). The exclusion criteria were: (1) participants with lower extremity injuries within 6 months; (2) participants who had or have AT injuries, MG strain and neurological diseases; (3) participants who had any training experience in any sports event and regular exercise habits (the average number of exercises per week was ≤1). The participants were forbidden from vigorous exercise within 24 h and warm-up before the test. All the participants provided written informed consent, and the study was approved by the ethics committee of Shanghai University of Sport (No. 2017007).

### 2.2. Measurement Procedure

#### 2.2.1. Architectural Properties of the gMTU

**Figure 1:**
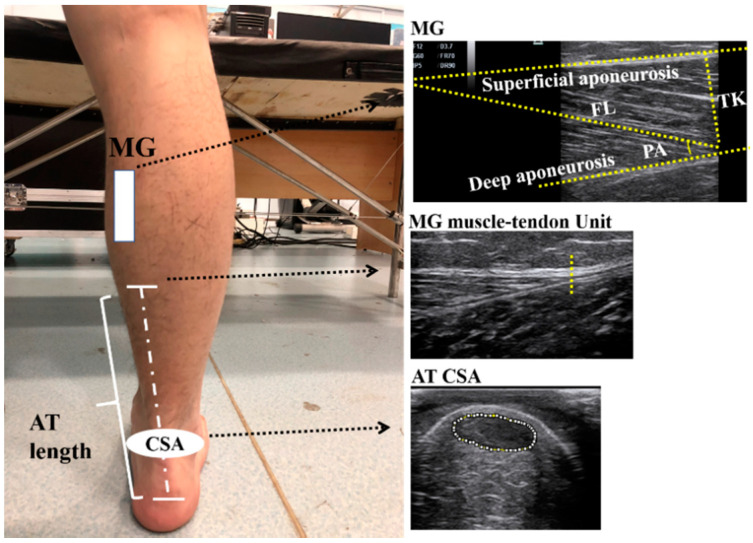
Diagram of the measurement of architectural properties and ultrasound images of the medial gastrocnemius (MG) and Achilles tendon (AT). Note: FL, fascicle length; PA, pennation angle; TK, thickness.

Shank length (from medial tibial condyle to the medial malleolus of the ankle) was assessed, first by using a measuring tape, and the participants were asked to seat with their ankle in neutral position and their knee and hip flexed at 90° at the same time [20]. After we determined the position of the MG belly (about 30% of the distance between the popliteal crease and the medial malleolus [21]), the participants were required to lie prone on the therapeutic bed with their ankles in a neutral position (the ankle should be perpendicular to the shank) and the muscle of the whole body relaxed. M7 Super ultrasonography system (Mindray, Shenzhen, China) with a linear array probe (L6-14) was used to determine the position of the AT insertion point at the calcaneus and the junction point of the MG and AT (Figure 1). The distance between these two points was defined as the AT’s length [22]. Afterwards, the AT’s CSA was measured at the horizontal position of the medial and lateral malleolus on the AT [23], and then the MG’s fascicle length, pennation angle and thickness were acquired at the MG belly of the dominant leg by the ultrasonographic system [21].

#### 2.2.2. Mechanical Properties of the gMTU

The vertical stiffness and logarithmic decrement (the dissipation of the mechanical energy) of MG and AT were measured by MyotonPRO (Myoton AS, Tallinn, Estonia) at the MG belly and 3 cm above the AT insertion point of the dominant leg [24]. The MyotonPRO probe needs to be vertical to the measuring area of the MG and AT whilst measuring.

All the devices we used in this study have been proved to possess reliability and to construct validity in previous studies already [25,26,27]. All the measuring procedures were executed by the same experimenter to avoid operation errors.

### 2.3. Data Processing

The ultrasound image was analyzed via ImageJ software (Version 1.53, NIH, Bethesda, MD, USA). The MG’s fascicle length was defined as the length of the fascicle between superficial and deep aponeuroses [21]. The pennation angle was the angle between the fascicle and deep aponeuroses and the thickness was the vertical distance between superficial and deep aponeuroses (Figure 1) [21]. The shallow, ellipse-like region in the ultrasound images is the CSA of the AT (Figure 1).

**Figure 2:**
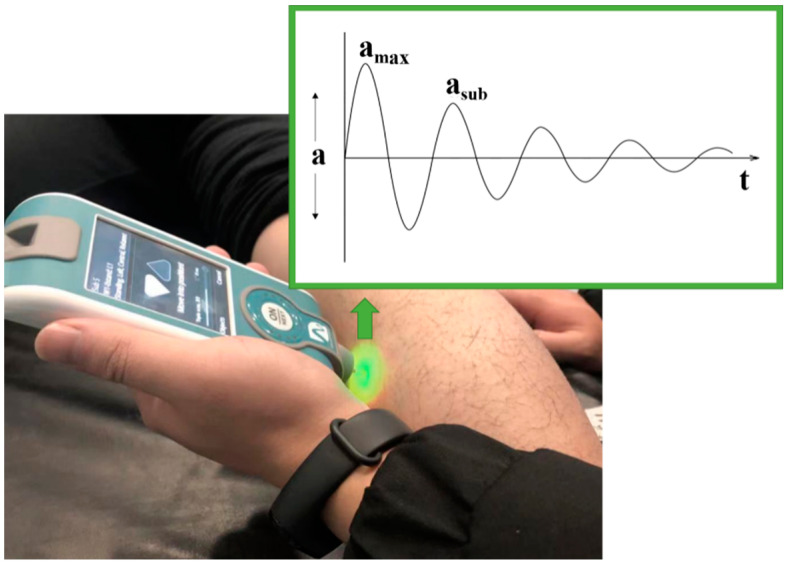
Diagram of the curve of damped natural oscillation measured by MyotonPro. Note: *a* is acceleration amplitude, *a*_max_ is the peak acceleration, *a*_sub_ is the submaximal acceleration.

The vertical stiffness and logarithmic decrement of AT and MG were acquired directly by the built-in algorithm of MyotonPro [28]. The equation for vertical stiffness is:

(1) $$S=\frac{\left(a_{\max}\cdot m_{\mathrm{probe}}\right)}{\Delta L}$$

where *S* is the vertical stiffness, *a*_max_ is the peak acceleration (Figure 2), *m*_probe_ is the mass of the probe, and Δ*L* is the distance the probe moved after touching the measuring point of the soft tissue.

Logarithmic decrement was calculated through the equation:

(2) $$D=\ln(\frac{a_{\max}}{a_{\mathrm{sub}}})$$

where *D* is the logarithmic decrement, *a*_max_ is the peak acceleration, and *a*_sub_ is the submaximal acceleration (Figure 2).

### 2.4. Statistics

A Shapiro–Wilk test was used to test the normality of all data distribution. An independent *t*-test was used to quantify the gender differences in the architectural and mechanical properties of the gMTU if the data were normally distributed. Otherwise, a Mann–Whitney U test was used. All results are shown as mean ± standard deviation. The effect size (Cohen’s *d*) of each parameter in this study was calculated. The significance level was set as 0.05.

## 3. Results

### 3.1. Gender Differences in the Architectural Properties of the gMTU

**Figure 3:**
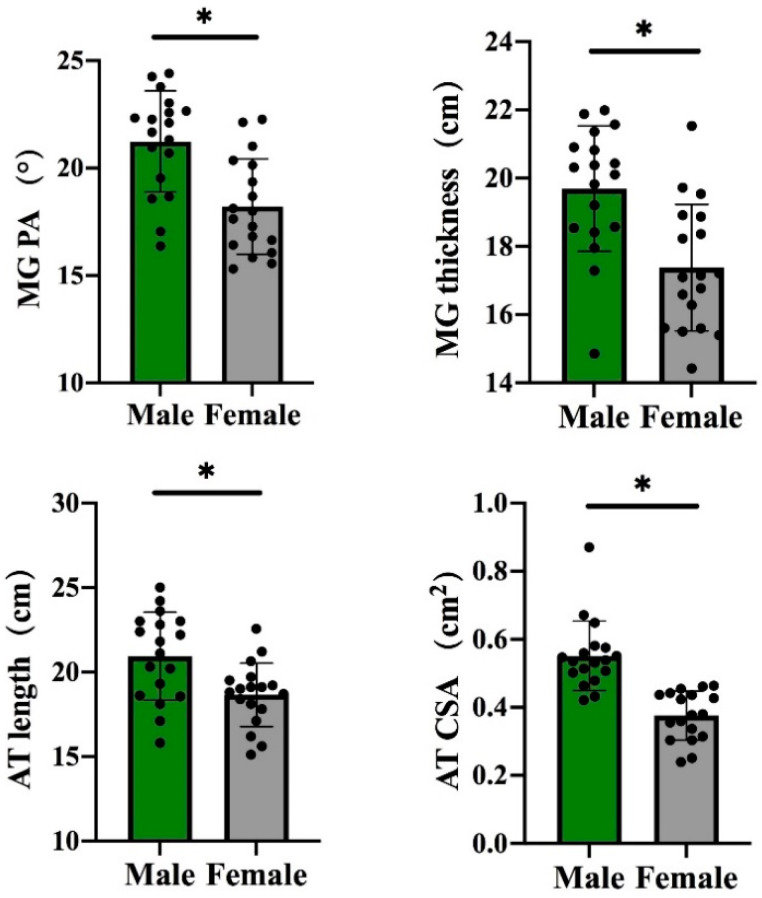
Differences in the architectural properties of the medial gastrocnemius (MG) and Achilles tendon (AT) between males and females. Note: PA, pennation angle; PCSA, physiological cross-sectional area; CSA, cross-sectional area; * indicates significant difference between males and females.

For individuals without any training experience and regular exercise habits, the MG’s architectural properties, namely, pennation angle and thickness, were greater in males than in females (*p* < 0.05, Figure 3), but no significant gender difference was observed in the MG’s fascicle length (Table 2). Moreover, the males possessed ATs with a longer length and a greater CSA than those of females (*p* < 0.05, Figure 3).

### 3.2. Gender Differences in the Mechanical Properties of the gMTU

**Figure 4:**
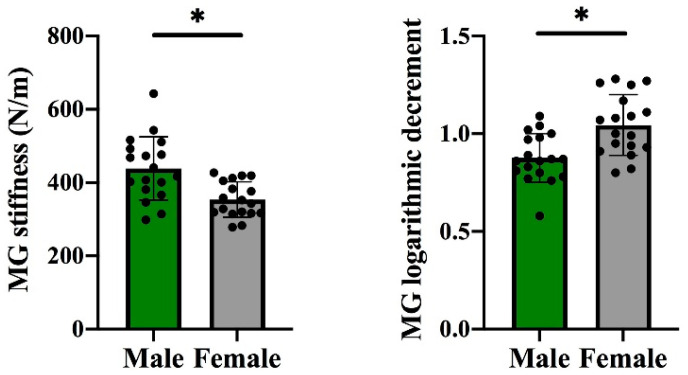
Differences in the mechanical properties of the medial gastrocnemius between males and females. Note: Stiffness indicates vertical stiffness, * indicates a significant difference between males and females.

For those without training experience and regular exercise habits, the MG’s vertical stiffness was lower and the MG’s logarithmic decrement was greater in females than in males (*p* < 0.05, Figure 4). However, no remarkable difference was observed in the AT’s vertical stiffness and logarithmic decrement between males and females (Table 2).

## 4. Discussion

In this study, we aim to explore the gender differences in the architectural and mechanical properties of the gMTU. As hypothesized, in terms of architectural properties, the MG’s pennation angle and thickness, as well as AT’s length and CSA, are longer or greater in males than in females. In terms of mechanical properties, females have a higher logarithmic decrement in MG. However, no substantial gender differences in MG’s fascicle length and AT’s mechanical properties were found.

In terms of architectural properties, the MG’s pennation angle and thickness were greater in males than in females. Unexpectedly, the fascicle lengths of the MGs between females and males were similar. The results were consistent with previous studies, which reported that males possess similar MG fascicle lengths but a thicker MG, with larger MG pennation angles compared with females [14,29,30,31]. Besides, previous studies also reported that it was significantly greater in the muscle thickness and pennation angle of the vastus lateralis and rectus femoris of males than females [32,33]. Leg length and architectural parameters have no correlation; therefore, the difference could reflect true gender-based differences [14]. Architectural differences might be caused by differences in testosterone levels, which strongly stimulate protein anabolism in muscle and causes more hypertrophy in the MG of men than that of women [34]. Moreover, the gastrocnemius is one of the biggest contributors to body weight support [3]. Therefore, the remarkable difference in weight between genders indicates the higher requirements of males for MG strength during the stance phase of walking, jumping or running, which leads to greater hypertrophy, compared with that of females. Furthermore, the architectural and mechanical properties of the MG could reflect physical function [7]. The architectural properties of the MG (e.g., pennation angle, thickness) are positively correlated to muscle strength, maximum force-developing ability, peak power, running economy and sprint performance [8,9,10,11,35,36,37,38]. Hence, the difference in architectural properties could partly explain the superior physical fitness, lower extremity strength, speed and performance in endurance-oriented sports events of males than females, because the greater MG force and power of males are beneficial for propulsion, as inferred from our results. Furthermore, muscle strength is a reliable biomarker for musculoskeletal health [39]. Thus, males might have better musculoskeletal health than females.

In terms of the mechanical properties of the MG, higher vertical stiffness and a lower logarithmic decrement were observed in males compared with females. These findings are consistent with the study of Blackburn et al. [40] The reason for the lower vertical stiffness in females might be due to higher oestrogen levels, which are related to collagen synthesis [41]. Furthermore, stiffer tissues could transmit force more rapidly [42], and a lower logarithmic decrement indicates higher elasticity with less mechanical energy dissipated [43]. Thus, the results inferred that the MG of males could produce a greater transit force more rapidly to the AT and waste less mechanical energy than that of females; hence, males have a better economy not only in daily activities but also in exercise. In the meantime, the higher vertical stiffness of MG in males might indicate that AT strain is lower [44]. Excessive muscle strain in the eccentric phase or stretching is the primary mechanism of muscle strain injury [45]; therefore, the risk of suffering from excessive muscle strain is lower in males than in females at an equal MG force.

Expectedly, the males possessed a longer AT and a greater CSA. Similarly, male athletes have greater tendon length and CSA than female athletes [46]. The longer AT in males could also be an implication for sports performance. Ueno et al. [38] found that a longer AT length is positively correlated with energy cost during submaximal running as it could store and return more elastic energy from the ground reaction force compared with a shorter AT. The greater AT CSA of males contributes to the potentially greater MG strength of males, as indicated by the higher AT force in males than in females [47]. Thus, the higher mechanical loads that can be exerted on the AT mean a greater adaptive hypertrophy because of increased collagen turnover in males. The results implied higher load-resisting capacities because the stress, a risk factor of AT injuries, is lower if equal force is exerted on the AT [47]. This result might suggest that the AT injury rate of males is probably higher than that of females. However, different from our hypothesis, no remarkable gender difference was observed in the mechanical properties of the AT. One explanation for this would be that the vertical stiffness we measured was perpendicular to the AT rather than along the AT, which is the direction with a continuous high load. Thus, the longer length and the greater CSA of the AT in males could store and return more elastic energy and withstand higher loads.

In this study, gender-related differences in the architectural and mechanical properties of the gMTU, particularly the physical fitness or performance deficits in females and the better musculoskeletal health and less vulnerability of males, were explained from the perspective of anatomy and biomechanics. Exercise/training methods (e.g., eccentric exercise/training) that can enhance the MG pennation angle and thickness and vertical stiffness could be further recommended to remedy the inferior physical fitness and exercise performance of females. However, this study has several limitations: (1) the vertical stiffness we measured was only perpendicular to AT rather than along the AT and further studies could calculate vertical stiffness using the AT force–tendon elongation relationship; (2) strength or performance was not tested to directly confirm the physical fitness and performance merits in males; and (3) both genders had no remarkable differences in height, weight and leg length in this study. However, leg length and muscle morphology have no substantial correlation [14]. Previous studies have stated that weight, height and leg length are not standardized. Therefore, we did not consider the influence of these factors on the gender difference in the gMTU’s morphological and mechanical properties.

## 5. Conclusions

Distinct gender-based differences exist in the architectural and mechanical properties of the MG and the architectural properties of the AT amongst Asian adults with irregular exercise habits. Specifically, among individuals without training experience and regular exercise habits, the MG’s pennation angle, thickness and vertical stiffness and the AT’s length and CSA are greater in males than in females. In addition, the AT’s logarithmic decrement is lower in males than in females. The MG’s force-producing capacities, ankle torque, mechanical efficiency and peak power are higher in males than in females. The load-resisting capacities of the AT might be greater and the MG’s strain is lesser in males than in females as well. These results potentially suggest that males have a lower AT and calf injury risk and better musculoskeletal health than females. Therefore, males have better physical fitness, speed and performance in power-oriented sports events than females from the perspective of morphology and biomechanics.

## Figures and Tables

**Table 1:** Basic information of participants.

Group	*n*	Age (years)	Height (cm)	Weight (kg)
Male	18	24.4 ± 2.1	175 ± 3.9	69.7 ± 7.3
Female	18	25.1 ± 1.6	161.6 ± 4.3	52.8 ± 5.4
*p*-Value		0.261	<0.001	<0.001

**Table 2:** Comparison of the shank length and the architectural and mechanical properties of the medial gastrocnemius and Achilles tendon between females and males.

	Shank Length (cm)	FL (cm)	PA (°)	TK (cm)	Vertical Stiffness _MG_ (N/m)	D_MG_	Length _AT_ (cm)	CSA (cm^2^)	Vertical Stiffness _AT_ (N/m)	D_AT_
Female	32.1 ± 1.7	5.7 ± 0.6	18.2 ± 2.2	1.7 ± 0.2	337.4 ± 89.8	1.0 ± 0.2	18.7 ± 1.9	0.4 ± 0.1	1205.4 ± 187.3	0.6 ± 0.3
Male	34.4 ± 1.9	5.9 ± 0.4	21.2 ± 2.4	2.0 ± 0.2	438.6 ± 86.4	0.9 ± 0.1	20.9 ± 2.6	0.6 ± 0.1	1228.6 ± 162.8	0.8 ± 0.3
*p*	0.001	0.226	<0.001	0.001	0.002	0.001	0.005	0.001	0.695	0.164
Cohen’s *d*	1.28	0.39	1.30	1.06	0.78	1.25	1.13	2.00	0.13	0.67

Note: MG is the medial gastrocnemius, AT is the Achilles tendon, FL is the fascicle length of the MG, PA is the pennation angle of the MG, TK is the thickness of the MG, D_MG_ is the logarithmic decrement in the MG, CSA is the cross-sectional area of the AT, and D_AT_ is the logarithmic decrement in the AT.

## Data Availability

The datasets generated during and analyzed during the current study are available from the corresponding author on reasonable request.
